# Recurrent TIPS dysfunction and variceal bleeding: A combined, staged, use of large-bore aspiration thrombectomy and partial splenic artery embolization—A case report

**DOI:** 10.1016/j.radcr.2024.10.003

**Published:** 2024-10-30

**Authors:** Dayoung Kim, Yejun Hong, Ani Mirakhur

**Affiliations:** a Cumming School of Medicine, University of Calgary, Calgary, Alberta, Canada; b Faculty of Diagnostic Radiology, University of Calgary, Calgary, Alberta, Canada

**Keywords:** Covered stent, TIPS, Splenomegaly, Splenic artery embolization

## Abstract

A 51-year-old man, with a history of cirrhotic portal hypertension and recurrent transjugular intrahepatic portosystemic shunt (TIPS) stenoses, presented with an acute gastro-esophageal variceal hemorrhage in the setting of an acute and massive thrombotic TIPS shunt occlusion. The clinical presentation was complicated by patient's severe, chronic thrombocytopenia which had precluded empiric anticoagulation previously for recurrent TIPS dysfunction. Following endoscopic treatment of the variceal bleeding, the CAT 12 Indigo aspiration system (Penumbra) was used to remove a large burden of thrombus from the TIPS, allowing successful re-stenting and restoration of blood flow through the TIPS. A staged partial splenic artery embolization was performed a few days later to reduce hypersplenism and treat the thrombocytopenia. The patient was then therapeutically anticoagulated to prevent future TIPS occlusion.

## Introduction

A TIPS conduit is designed to bridge the portal and hepatic venous circulation to manage portal hypertension, thereby treating complications such as variceal hemorrhage and ascites [1]. A common and important complication of this therapy is thrombotic shunt occlusion, of which rates have improved with the use of expanded polytetrafluoroethylene grafts [2]. The traditional options for managing shunt thrombosis include catheter-directed thrombolysis (CDT) and systemic anticoagulation [3]. However, systemic anticoagulation for prophylaxis or treatment of TIPS thrombosis is contraindicated if the patient carries significant risk of excessive hemorrhage. Therefore, in this case, mechanical thrombectomy (MT) was employed instead, which has been previously described but only with older generation thrombectomy devices [4].

Another concern with patients with liver disease advance enough to warrant TIPS is thrombotic dysregulation. Thrombocytopenia in cirrhotic patients (platelets <50,000/μL) is associated with increased variceal bleeding and dysregulation of the coagulation pathway [5], which can be mitigated through pharmacological or surgical means [6]. Unfortunately, most of these patients have extensive burden of portosystemic shunts and end-organ varices which preclude surgical options. Partial splenic artery embolization (PSE) is a minimally invasive procedure that can effectively improve liver function and platelet counts, and has been associated with fewer postoperative complications than a splenectomy [7].

We describe the case of a 51-year-old man with acute, massive, TIPS thrombosis in the setting of variceal hemorrhage and chronic, severe thrombocytopenia where use of the Indigo CAT 12 large bore aspiration thrombectomy allowed for safe removal of large volume thrombus. A staged PSE was subsequently performed to improve serum platelet count to facilitate safe indefinite anticoagulation.

## Case report

**Fig. 1:**
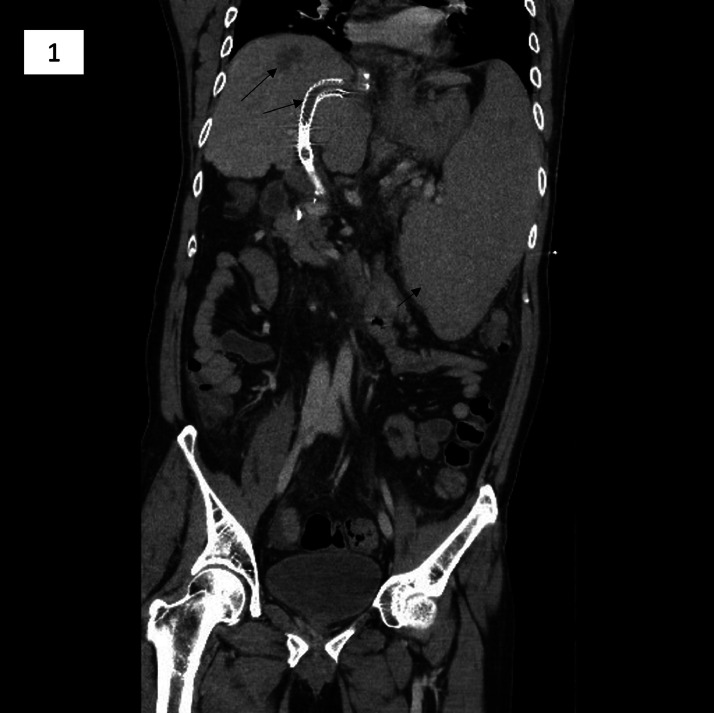
Coronal CT image shows thrombotic occlusion of the TIPS shunt and massive splenomegaly. Note hepatic dome hypodense lesion which represents post ablation changes related to a hepatocellular carcinoma.

A 51-year-old man with Child Pugh C cirrhosis presented to the ER with large volume gastrointestinal (GI) bleed and hypovolemia (vital signs showed T(C) 38.6°C; BP 104/73; Pulse 115 bpm; RR 36/min; SpO2 96%). The patient was pale with cold and clammy extremities, with altered mentation. His breath sounds were clear and abdomen was nondistended. His INR was 1.9, platelet count was 30×10^9^/L, and bilirubin was 27 μmol/L with otherwise normal liver panel. The patient's medication included carvedilol 6.5 mg daily, furosemide 20 mg daily, spironolactone 50mg daily, rifaximin 550 mg BID, and daily lactulose 15-30mL, to which he was adherent. Patient was not a candidate for liver transplantation due to multiple comorbidities and history of multifocal HCC treated with 3 sessions of thermal ablation. He had TIPS placed 7 years ago for secondary prevention of variceal bleeding. Since then, the patient had at least 5 TIPS revision procedures (angioplasty/thrombectomy) to treat in-stent stenoses. This issue stemmed from a lack of preventative anticoagulation, due to the patient's severe spleen-induced thrombocytopenia (Fig. 1).

An abdominal CT on this visit revealed massive thrombosis within the TIPS shunt, on a background of chronic superior mesenteric vein thrombosis. His variceal bleed was managed endoscopically with banding, after which interventional radiology service was involved for TIPS thrombectomy.

**Fig. 2:**
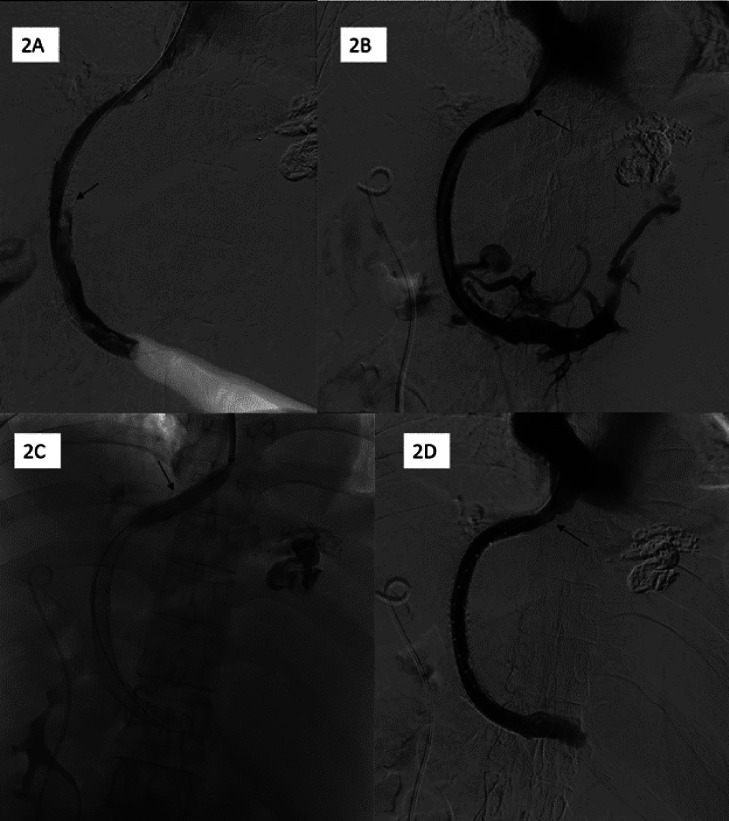
DSA images from the TIPS thrombectomy procedure. (A) demonstrates residual thrombus (arrow) in the TIPS shunt after a few passes of suction thrombectomy. Relining the TIPS shunt was successful after thrombectomy, and a stenosis in the hepatic vein just cephalad to the TIPS shunt can be seen (arrows) (B). 10 mm Balloon angioplasty was therefore performed (C). After the procedure, the shunt is widely patent with improved caliber of the hepatocaval junction stenosis (D).

The thrombosed TIPS conduit was cannulated via a transjugular approach with minimal difficulty. Because of the significant amount of clot within the TIPS stent, mechanical thrombectomy was performed, using the Indigo Lightning 12 (Penumbra Inc., Alameda, CA) catheter. Multiple passes were made, and large volume of organized thrombus was removed (Fig. 2A). At this point, a fibrotic stenosis was unmasked at the stent's hepatic venous end (Fig. 2B). Having debulked a majority of the in-stent thrombus, the TIPS conduit was relined with Gore Medical's Viatorr CX 8-10 mm x 7 cm x 2 cm stent-graft, taking care to cover the hepatic venous end stenosis. Final portal angiogram after 10 mm balloon angioplasty (Fig. 2C) showed a functioning TIPS shunt, though the portosystemic venous gradient remained elevated at 30 mmHg (Fig. 2D).

**Fig. 3:**
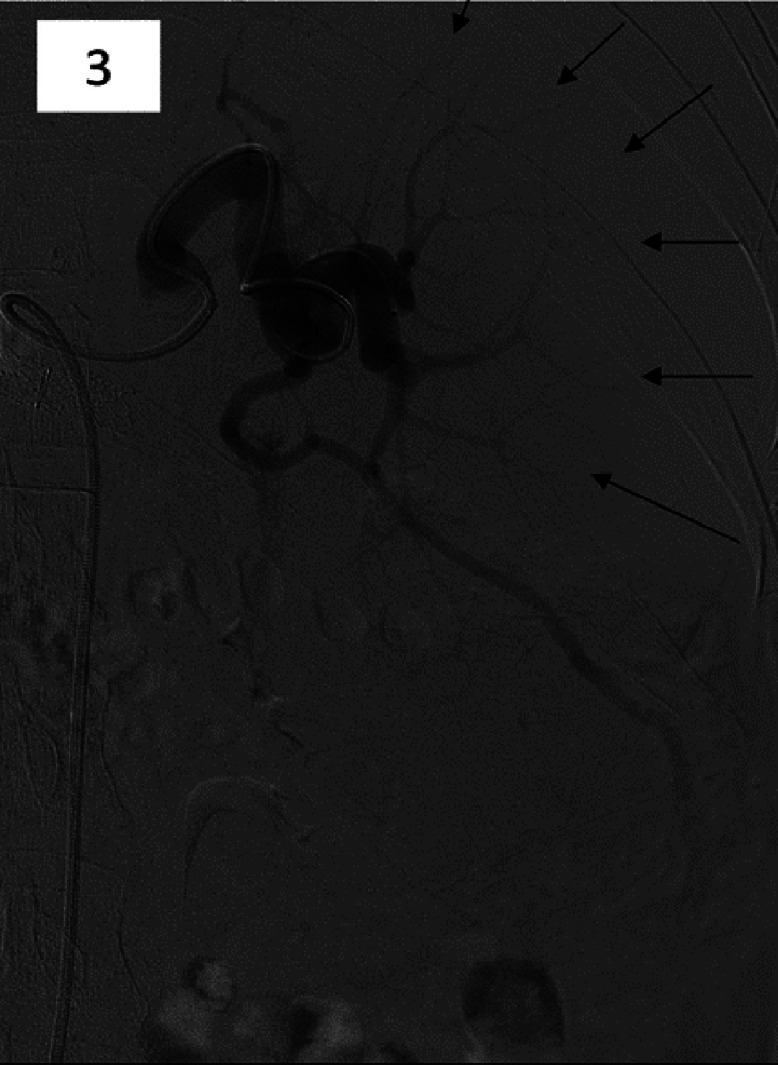
4 days after the TIPS thrombectomy, partial splenic artery embolization was successfully performed with particles and coils to treat hypersplenism (arrows).

72 hours later, the patient underwent a PSE to treat the splenomegaly. The PSE was performed via femoral access and involved subselective catheterization of splenic artery branches and embolization with 700–900-micron embosphere (BioSphere Medical S.A.) particles to infarct approximately 50% of the splenic parenchyma (Fig. 3). Care was taken to avoid embolization of upper pole branches. There were no intraprocedural vascular complications, and thus manual compression was sufficient to achieve hemostasis. After the thrombectomy, platelet and octreotide infusions were initiated to lower the bleeding risk. The patient tolerated the procedure well and needed narcotic analgesia for a few days post procedure. His platelet count improved from a nadir of 24×10^9^/L preprocedure to a peak of 191×10^9^/L approximately 8 days following the procedure. He was started on indefinite therapeutic anticoagulation and did not experience any further upper or lower gastrointestinal bleeding. A follow-up abdominal ultrasound 2 weeks later confirmed wide patency of the revised TIPS conduit with satisfactory flow velocities. He was discharged from the hospital a month later.

## Discussion

Shunt stenosis or thrombosis is a common complication after TIPS insertion with literature citing that 70-90% of patients require reintervention within 2 years [8]. This data is concordant with our clinical scenario, as the patient underwent multiple revisions. These are challenging patients to manage as anticoagulation strategies need to be balanced against the high risk of bleeding. In our patient, GORE® VIATORR® TIPS Endoprosthesis was used, which is a covered stent. The recurrent stent thrombosis despite this equipment was attributed to the inability to employ anticoagulation in the context of chronic severe thrombocytopenia.

There have been other studies that demonstrated effective application of mechanical thrombectomy. Molano et al. had successfully performed large-bore thrombectomy with the FlowTriever System [9]. Mechanical thrombectomy was also effective for noncirrhotic patients with portal venous thrombosis. In a study by Li et al., 23 cases of thrombectomy were reviewed, where 5-fr aspiration catheter was used in noncirrhotic patients with no serious complications [10]. Our case is different from the above studies in that the large-bore Indigo system was applied to remove large thrombotic burden in a cirrhotic patient with significant coagulopathy, with no unforeseen complications. These articles also show that mechanical thrombectomy is a safe and effective method in immediately re-establishing portal venous blood flow, thus preventing further vascular complications of cirrhosis.

Therapies for TIPS thrombosis include systemic anticoagulation, catheter-directed thrombolytic therapy, mechanical thrombectomy, and liver transplantation. A combination of mechanical thrombectomy and pharmacologic thrombolysis are often used together via catheter access [11]. Treatment options for this patient were limited to mechanical thrombectomy, because the severe thrombocytopenia (30×10^9^/L) and elevated INR precluded pharmacologic thrombolysis, and the patient declined liver transplantation. During the procedure, the Lightning 12 system, with its large lumen, was effective at large-volume thrombus removal from the TIPS shunt with minimal blood loss. This allowed for a successful relining of the TIPS shunt without incurring risk of hemorrhage or iatrogenic pulmonary embolization.

Other aforementioned therapies to address TIPS thrombosis have risks and benefits. Systemic anticoagulation inhibits the coagulation cascade to prevent clots from propagating, but cannot recanalize through preexisting thrombi. In the case of an obstructive TIPS thrombus, persistent blockage may result in sequelae of portal hypertension such as cavernous transformation of the portal system or progression of variceal bleeding [11]. Thrombolytic therapy is another effective method that actively breaks down the fibrin matrix in clots. However, there is also an increased systemic risk of bleeding, and therefore increased INR and severely diminished platelet count precludes this therapy [12]. Lastly, while liver transplantation is a curative therapy to cirrhosis and hepatogenic coagulopathy, it is highly invasive and is associated with multiple short- and long-term complications, from opportunistic infections to acute cell rejection and even hepatic artery thrombosis [13].

After the mechanical thrombectomy, the patient received partial splenic artery embolization (PSE). PSE is a catheter-guided intervention that addresses hypersplenism secondary to chronic liver disease. Embolizing some of the splenic artery branches infarcts splenic tissues, which in turn shrinks the spleen and reduces portal vein pressure. Literature has shown that partial splenic artery embolization improves liver function, pancytopenia, and coagulopathy in cirrhotic patients [14]. Further, the resolution of thrombocytopenia allows for anticoagulation, leading to prevention of thrombus formation.

While the above combination of mechanical thrombectomy and partial splenic embolization appropriately addressed this patient's medical needs, this is a single case report, which presents a challenge regarding the generalizability of the outcome. Risk and benefits of each therapy must be individually considered for every patient.

## Conclusion

Acute TIPS thrombosis in the setting of variceal bleeding is a challenging complication as the risk of bleeding needs to be balanced against the need to restore shunt patency from thrombotic occlusion. This case highlights a successful use of large-bore aspiration thrombectomy for large volume thrombus extraction from the TIPS conduit, offering an effective alternative to resolve stent thrombus when anticoagulation is contraindicated. This was followed by a staged PSE to treat chronic thrombocytopenia and allow for therapeutic anticoagulation to prevent future occlusion.

## Declaration of generative AI and AI-assisted technologies in the writing process

During the preparation of this work, the authors did NOT use any Generative AI or AI-assisted technology throughout the writing process.

## Patient consent

This work was carried out in accordance with the Declaration of Helsinki. Informed consent was obtained for publication of this case report.
